# Can We Increase the Resection Rate by Minimally Invasive Approach? Experience from 100 Minimally Invasive Esophagectomies

**DOI:** 10.1155/2019/3809383

**Published:** 2019-02-24

**Authors:** Olli Helminen, Johanna Mrena, Eero Sihvo

**Affiliations:** Department of Surgery, Central Finland Central Hospital, 40620 Jyväskylä, Finland

## Abstract

**Background:**

Whether we can increase the resection rate of esophageal cancer by minimally invasive esophagectomy (MIE) is unknown. The aim was to report the number and results of MIE in high-risk patients considered unsuitable for open surgery and compare these results to other operated patients and to high-risk patients not undergoing surgery.

**Methods:**

At Central Finland Central Hospital, between September 2012 and July 2018, the number of operated MIEs was 100. Of these, 10 patients were prospectively considered unfit for open approach. Nineteen additional high-risk patients with operable disease were ruled out of surgery. The short- and long-term outcomes of these 3 groups were compared.

**Results:**

In patients eligible for any approach (n=90), MIE only (n=10), and no surgery (n=19), WHO performance status Grade 0 was observed in 66.7%, 20.0%, and 5.3%, respectively; stair climbing with ≥4 stairs was successfully completed in 77.8%, 50%, and 36.8%, respectively. Between any approach and MIE only groups, rate of major complications (Clavien-Dindo ≥3a) was 6.7% vs. 50.0% (p<0.001) without a difference in median hospital stay (9 vs. 10 days, p=0.542). Readmission rates were 4.4% vs. 30.0% (p=0.003). Survival rates were 100% vs. 80% (p<0.001) at 90-days, 91.5% vs. 66.7% (p=0.005) at 1-year, and 68.9% vs. 53.3% (p=0.024) at 3-years, respectively. In comparison between MIE only and no surgery groups, these survival rates from day of diagnosis were 80% vs. 100%, 68.6% vs. 67.1%, and 45.7% vs. 32.0% (p=0.290), respectively.

**Conclusions:**

By operating patients unsuitable for open approach with MIE, the resection rate increased 11.1%. These high-risk patients had, however, higher early morbidity and reduced long-term survival compared to other operated patients. Though there seems to be long-term benefit of surgery compared to nonsurgical patients, we have to be cautious when offering surgery to those considered unfit for open surgery.

## 1. Introduction

Worldwide esophageal cancer is the sixth leading cause of cancer death [1]. For cure, surgery offers the best chance in early or locally advanced disease [2], where modern multimodal treatment protocols have improved survival rates [3]. Currently, 5-year survival rates at the population level are around 45% after surgery compared to dismal 4-6% among patients considered unfit for surgery or with advanced disease [4, 5].

In order to reduce surgery-associated morbidity and mortality, minimally invasive esophagectomy (MIE) has been introduced [6]. This approach seems to reduce postoperative morbidity, mortality, and hospital stay and improves patient satisfaction [6–10]. Improved 3-year overall survival has been reported after MIE in a randomized trial, although without statistical significance, suggesting at least comparable oncologic outcome [11]. Nowadays, at many specialized centers, majority of esophagectomies can be performed with minimally invasive approach [12, 13]. Less invasive surgery with proven advantages could even be considered for high-risk patients unsuitable for open surgery. At the population level in Finland and Sweden, the era of implementing MIE to clinical practice has, however, not increased the resection rate of esophageal cancer, which has remained around 30% since year 2000 [5, 10]. In England, the resection rate is as low as 18.2% [14].

The aim of the current study was to evaluate the number and the results of MIE for high-risk patients with esophageal cancer considered preoperatively unsuitable for open surgery at a specialized center with previously reported excellent surgical results [13, 15]. Outcomes were compared to those of patients suitable for any surgical approach and to high-risk patients with potentially curative disease not undergoing surgery.

## 2. Materials and Methods

### 2.1. Patients

In September 2012, the MIE program at Central Finland Central Hospital was started by an experienced surgeon (ES) [15]. After a gradual start, the annual caseload has been 20 to 25 operations. All cancer operations of the tubular esophagus or the esophagogastric junction (n=100) between September 2012 and July 2018 have been performed using either totally minimally invasive or hybrid approach (Tables 1 and 2). In thorough preoperative evaluation registered in prospective database, 10 patients (10%) were classified to have a very high surgical risk or an extended surgical indication and were, therefore, considered unsuitable for open surgery (Table 3). Additionally, total of nineteen patients with potentially curative local or locally advanced disease did eventually not undergo surgery (Table 4).

The preoperative diagnostic and staging protocol included endoscopy, endoscopic ultrasound, body computed tomography (CT), and positron emission tomography- (PET-) CT. Also, patients' exercise tolerance with stair climbing test and nutritional status was routinely evaluated [16]. The patients' baseline information is provided in Table 1. Of 100 patients, 77 received neoadjuvant therapy, including either chemotherapy or chemoradiation. The intended chemotherapy cycle consisted of a single dose of epirubicin (50 mg/m^2^) and cisplatin (60 mg/m^2^), and 5-fluorouracil 200 mg/m^2^/day for 21 days. Three cycles were given preoperatively and three postoperatively. Chemoradiotherapy included paclitaxel (50 mg/m^2^) and carboplatin (180-300 mg) for four cycles and 23 fractions of radiation for a total of 41.4 Gy. Patients were restaged before surgery with either CT or PET-CT according to primary fluorodeoxyglucose (FDG) avidity of the tumor. Only high FDG-avid tumors were restaged by PET-CT. The operation was performed approximately after a 6-week recovery period, depending on the reevaluation of the physical condition. The Charlson comorbidity index was calculated from existing comorbidities excluding the esophageal cancer under treatment [17].

### 2.2. Operative Approach

In 92 patients, the planned operation was transthoracic total MIE and in 8 patients a hybrid procedure, with either chest (n=5) or abdomen (n=3) performed using an open approach. The reasons for the planned thoracotomies were T3-tumor location against the main bronchi (n=4) or mediastinal inflammation caused by stent penetration (n=1). The need for colon interposition (n=2) and severe adhesions after peritonitis (n=1) were the reasons for planned laparotomies. In addition, two were converted to a hybrid procedure due to a short gastric conduit after a previous fundoplication or severe adhesions in the abdomen after peritonitis. Intrathoracic anastomosis was our preference (n=88). Neck anastomosis was performed in 12 patients. Colon interposition was used in 2 patients, one with neck anastomosis due to recurrence after previous MIE with intrathoracic anastomosis and the other with thoracoscopic intrathoracic anastomosis after previous gastric sleeve resection, pancreatitis, and necrosectomy. All patients underwent en bloc lymphadenectomy with 3-dimensional optics used since June 2013. Perioperative standardized treatment protocol, extent of lymphadenectomy, and follow-up have been previously described [13, 15]. The median follow-up time was 21 (IQR 11-38) months. Mortality data was confirmed from the nationwide and obligatory Cause of Death registry held by Statistics Finland. The end of follow-up for this study was November 5, 2018.

### 2.3. Reporting of Complications

The complications basic platform published by the Esophagectomy Complications Consensus Group (ECCG) [18] was strictly used. Overall, minor and major complications were reported according to Clavien-Dindo classification [19]. Positive resection margin, the number of examined lymph nodes, 30- and 90-day comprehensive complications index [20], and 30- and 90-day and 1- and 3-year mortality rates were reported.

### 2.4. Pathological Analysis

Paraffin-embedded esophageal samples were analyzed by a gastrointestinal pathologist according to the normal standardized protocol. Staging was performed according to the American Joint Committee on Cancer, seventh edition criteria [21].

### 2.5. Statistical Analysis

Baseline characteristics were analyzed using Chi-square or Mann-Whitney U tests as appropriate. Kaplan-Meier survival curves were calculated according to the life table methods to visualize the crude all-cause mortality rates. Statistical significance was assessed with log-rank test. Complications according to Clavien-Dindo were reported up to 30 days after surgery, and with comprehensive complications index separately at 30 and 90 days after surgery. Readmission rate was reported at 30 days after discharge. All analyses were conducted using the statistical software IBM SPSS 25.0 (IBM Corp., Armonk, NY, USA).

### 2.6. Ethical Statement

The study was approved by the Central Finland Hospital District.

## 3. Results

### 3.1. Basic Characteristics of Study Patients

Among those 119 potentially operable patients with local or locally advanced esophageal cancer referred to our center, the resection rate was 84.0% (n=100). The median (IQR) age of these operated patients was 68 (59-72) years, with a male majority (75/100). WHO performance status >0 was recorded in 38/100, and significant comorbidities were recorded in 48/100 patients. Adenocarcinoma was more common histology (79/100). The majority of malignant tumors were located at the distal esophagus or at the esophagogastric junction (94/100). Of 100 operated cancer patients, 77 received either neoadjuvant chemotherapy or chemoradiotherapy. Complete response was observed in 20 patients. Tumor stage and basic characteristics are listed in Table 1.

### 3.2. Comparison of Preoperative Parameters between Study Groups

In three study groups of any approach, MIE only, and no surgery mean age (SD) was 65.7 (9.4), 71.1 (10.2), and 73.2 (12.1) years, respectively. Median values are presented in Table 1. Most significant differences between any approach and MIE only groups were observed in physical performance. WHO performance status (p<0.001) and exercise capacity (77.8% vs. 50.0% climbed 4 or more staircases, p=0.004) were worse in MIE only group. MIE only patients, despite of similar rate of locally advanced tumors, received also less often preoperative oncological treatment (82.2% vs. 50.0%, p<0.001) (Table 1). Two patients with esophageal adenocarcinoma in the MIE only group were originally diagnosed with liver metastases. They had complete and a lasting liver response after chemotherapy (epirubicin, oxaliplatin, capecitabine, EOX) followed by local radiotherapy. One had persistent disease and the other one recurrence in the esophagus requiring further therapy. Patients had MIE two and six years after diagnosis of metastatic liver disease, respectively. Of these two, one died 13 months after surgery due to recurrent disease, and the other had recurrence 16 months after MIE.

Patients not suitable for surgery had limited exercise capacity (36.8% climbed 4 or more staircases), worse performance status (WHO Grade II in 42.1% of patients), and often a reduced pulmonary function (median FEV1 71%) when compared with other groups (Table 1). Of these 19 patients, 7 (36.8%) were referred to neoadjuvant treatment but were later conclusively ruled out of surgery (Table 4). Of these, 2 patients considered for surgery had a complete response and are, therefore, under careful surveillance without recurrence 21 and 65 months after diagnosis. Definitive chemoradiotherapy or palliative radiotherapy was given to 11 (57.9%) (Table 4).

### 3.3. Postoperative Outcomes

Any morbidity at 30 days after surgery was observed in 42.2% in any approach group and 70% in MIE only group (p=0.094). Between these groups, a significant difference was detected in the rate of major complications (Clavien-Dindo ≥3a), 6.7% vs. 50.0%, p<0.001, Table 2. Of these in any approach group, two were intubation injuries, two were pleural fluid collections treated with repeated punctures, one was type 2 anastomotic leak, and one was empyema treated with thoracoscopy and decortication. None of the patients in any approach group died during 90 days. In contrast, two patients (20%, p<0.001) died in MIE only group. One with cardiomyopathy faced a sudden death in the early morning of the planned discharge day (postoperative day 9). Of two anastomotic leaks (types 2 and 3), patient with type 3 leak died at postoperative day 42. Of the two patients with child A cirrhosis, one developed chylothorax, and the other developed pneumonia and pneumothorax on postoperative day 10. Both were treated by pleural drainage.

No difference was observed between any approach and MIE only groups in the median length of hospital stay (9 vs. 10 days, p=0.542). The rate of ICU (0 vs. 30.0%, p<0.001) and hospital readmissions (4.4% vs. 30.0%, p=0.003) were significantly higher in MIE only group.

### 3.4. Survival

**Figure 1:**
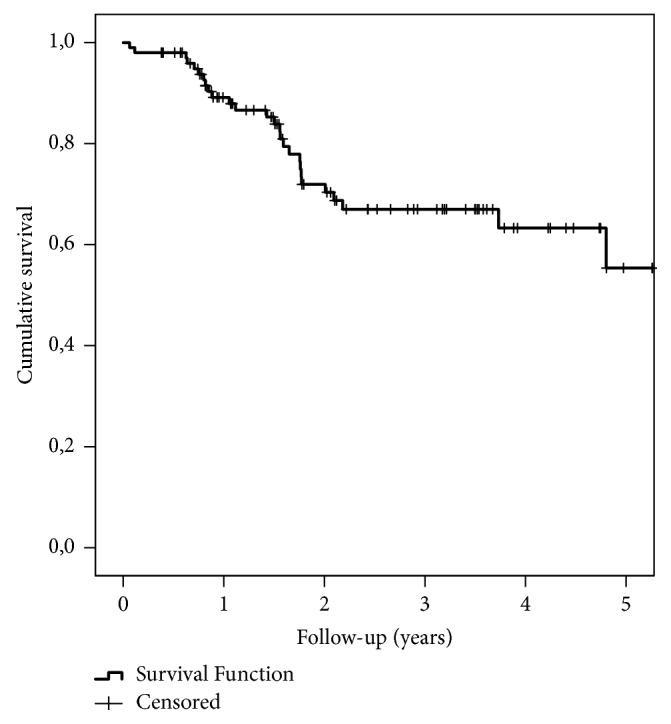
Kaplan-Meier survival curve of all 100 patients undergoing MIE. The starting point is the day of surgery.

**Figure 2:**
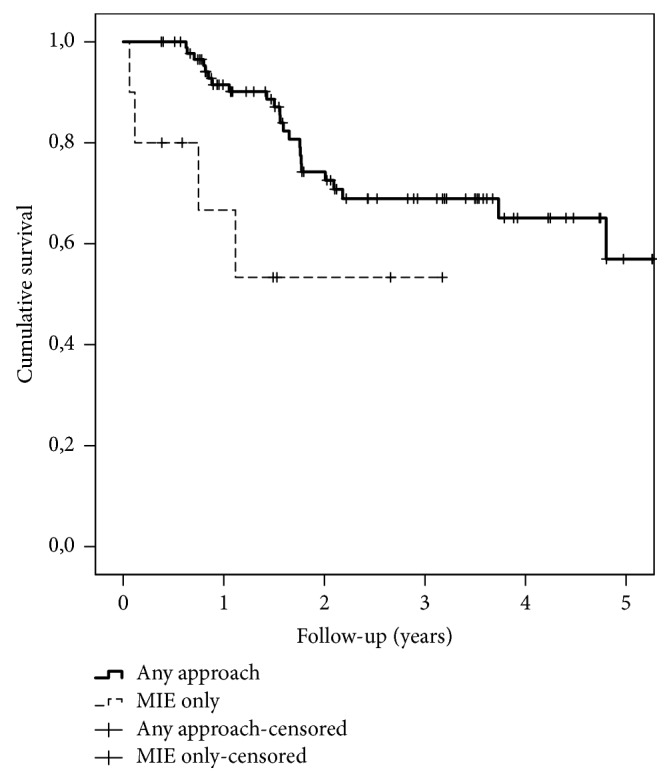
Kaplan-Meier survival curves of 90 patients (solid line) eligible for any approach and 10 patients fit for MIE only (dotted line). The starting point is the day of surgery.

In Kaplan-Meier analysis of all operated 100 patients, 90-day survival was 98.0%, 1-year survival 89.1%, and 3-year survival 67.0% (Figure 1). The differences in survival between any approach and MIE only groups during the whole follow-up were statistically significant (p=0.024): at 90 days 100% vs. 80.0% (p<0.001), at 1-year 91.5% vs. 66.7% (p=0.005), and at 3 years 68.9% vs. 53.3% (p=0.024), respectively (Figure 2).

**Figure 3:**
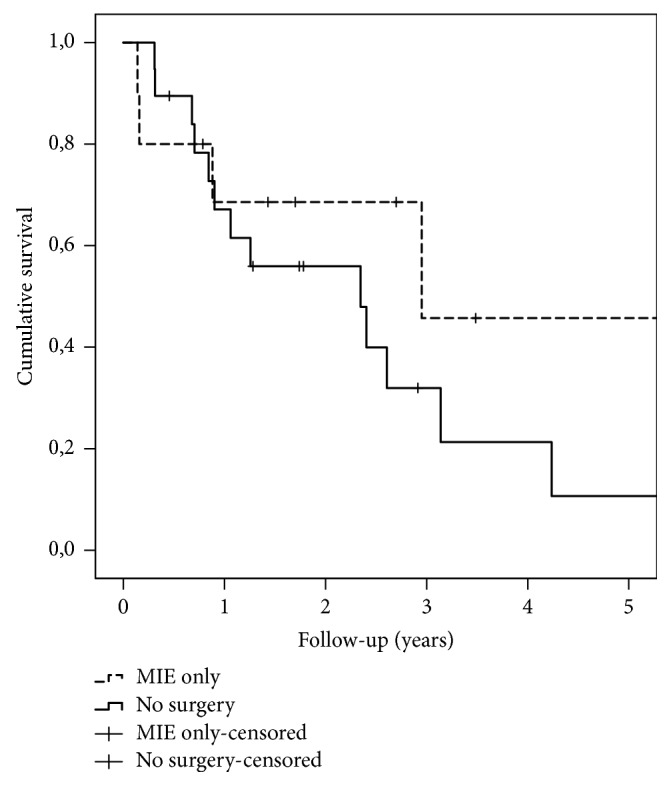
Kaplan-Meier survival curves of 10 patients (dotted line) fit for MIE only and 19 patients ruled outside surgical intervention (solid line). The starting point is the day of diagnosis.

In comparison of survival between MIE only and no surgery groups, the day of diagnosis was used as the start of the follow-up. Between these groups, no significant difference existed in survival during the whole follow-up (p=0.290). At 90 days, survivals were 80% vs. 100% (p=0.045), at 1-year 68.6% vs. 67.1% (p=0.809), and at 3 years 45.7% vs. 32.0% (p=0.863), respectively (Figure 3).

## 4. Discussion

In the present study, MIE increased the resection rate of esophageal cancer by 11.1%. Among these additional high-risk patients major complications were, however, more common and the risk of short- and long-term mortality was higher than in patients suitable for open surgery. Short-term survival of operated high-risk patients was also worse compared to patients who underwent no surgery. The absolute benefit of surgery in these high-risk patients was 13.7% at 3 years.

Strengths of our study are completed learning curves, shown to affect outcomes [22–24], and excellent and stable reported results [13]. Therefore, technical difficulties or variations in the operations are minimal, and differences between groups in outcomes can be assumed to be mainly due to patient-specific factors. Nationwide compulsory databases enabled us to receive complete long-term survival data. The major weakness of our study is the relatively small number of high-risk patients. Preoperative risk evaluation and grouping with prospective data collection reduced, however, the risk of selection bias. Furthermore, all high-risk patients were evaluated by a single surgeon providing a homogenous setting in the preoperative workup. Because patients have heterogeneous reasons for increased surgical risks, we were unable to provide simple cut-off values to high-risk grouping.

The overall 90-day mortality rate of 6 to 9% after esophageal cancer surgery is often seen at the population level [10, 25, 26]. With such a mortality and with significant morbidity rates many patients with resectable disease either are not offered surgery or decline surgery causing differences in utilization of surgery for esophageal carcinoma between countries and even between the areas of one country [14, 27, 28]. At a specialized referral center, the resection rate is significantly higher [29]. Our resection rate in potentially resectable disease was 84% being higher than the rate of 64% at a specialized referral center at New York State [29]. In population-based studies, overall resection rates have varied from 18.2% and 18.7% in England [14] and New South Wales [30] to 29.9% in Denmark [14]. Regardless of those high-risk patients in our series increasing the resection rate, the overall 90-day mortality of 2.0%, 1-year survival of 89.1%, and 3-year survival of 67.0% are comparable to those reported, respectively, as 2.4%, 85.5%, and 62.2% in the low-risk benchmarking series of MIE [12]. These kinds of results justify surgery for high-risk patients. With the reported good overall outcomes and after completed learning curve of MIE, extending surgery to very high-risk patient not considered suitable for open surgery seems possible.

The high early mortality and morbidity rate in the MIE only group raises the following question: what is the benefit of surgery in these borderline patients? The comparison of survival between MIE only and no surgery groups is somewhat convoluted due to differences in risk profile, histology, and response to neoadjuvant therapy. Two patients with complete neoadjuvant response and higher rate of squamous cell cancers responding well to chemoradiotherapy improve survival in no surgery group [31]. On the other hand, of 19 nonsurgical patients, 14 were eventually physically unfit for surgery. Of these, all except one patient received, however, oncological treatment. Therefore, a reasonable comparison between groups was considered possible. The absolute benefit of surgery was 13.7% (45.7% vs. 32.0%) at 3 years. Though the difference was not statistically significant, it seems that those patients with life expectancy more than just 1 to 2 years would benefit of surgery. According to our data and previous results [32], the completion of multimodality treatment is extremely difficult in high-risk patients with a high drop-out rate [32]. Therefore, elderly and frail patients should selectively undergo upfront surgery, chemoradiation therapy, or palliative care.

The increasing burden of cancer in aging population associated with other comorbidities has become a prominent issue in cancer surgery [1]. Compared to esophageal cancer patients operated in Finland and Sweden with the mean age of 64.8 years, the mean age in this study in any approach group was 65.7 years and in MIE only group 71.1 years. Age and comorbidities, predictors of major morbidity and mortality after esophagectomy, have independently impact on the resection rate and survival [33–36]. For example, in elderly patients (>80 yrs), surgery is rarely used [37, 38]. Specific comorbidities or even combinations of these estimate, however, poorly the surgical mortality [39]. Therefore, it is difficult to set any cut-off points to decline surgery in specific known risk-factors or in their combination. For example, liver cirrhosis, being not an absolute contraindication for esophagectomy, has often been considered as such [40]. Furthermore, though the benefits of minimally invasive approach in high-risk patients are evident, the risk estimation studies in esophageal cancer surgery are done during the era of open surgery [41–43].

Salvage esophagectomy after previous definitive chemoradiotherapy is a well-established treatment strategy [44]. Any role of surgery in stage IV disease can be disputed. Previously, a German series of 70 patients diagnosed with metastatic esophageal adenocarcinoma undergoing surgery of the primary tumor and metastasis reported good survival outcomes especially if a good response to chemotherapy was achieved [45]. In one case report an esophageal cancer patient with liver and lung metastases had a complete chemotherapy response and underwent esophagectomy 4 years later due to a local recurrence [46]. In our study, two patients were originally diagnosed with liver metastases but had complete and lasting liver response with chemotherapy. They developed local recurrence in the esophagus regardless of radiotherapy and were eventually operated by MIE. In this kind of rare occasion with uncertain surgical outcome, patients and oncologists have a lower threshold to referral to MIE compared to open surgery. The survival benefit of surgery is, however, unproven.

The findings in this study could have clinical implications. As MIE is becoming more commonly used technique with more surgeons completing learning curves, surgical treatment can be offered increasingly to high-risk patients and with extended indications. Because of the relatively high morbidity and mortality related even to MIE, more studies are needed to assess the short- and long-term outcomes of surgery and compare those to various other treatment modalities in high-risk patients.

## Figures and Tables

**Table 1:** Patient characteristics.

	Fit for any approach n=90	Fit for MIE only n=10	No surgery n=19	P value: Any approach – MIE only	P value: Any approach – No surgery	P value: MIE only – No Surgery
Age yrs (median, IQR)	68 (59-72)	70 (59-83)	73 (61-86)	0.172	*0.014*	0.359

BMI kg/m^2^ (median, IQR)	25.0 (22.5-28.3)	28.9 (22.3-35.7)	20.8 (19.6-no data)	0.111	0.065	0.116

Male, n (%)	68 (75.6)	7 (70.0)	12 (63.2)	0.700	0.266	0.713

WHO performance status, n (%)				*<0.001*	*<0.001*	0.306

Grade 0	60 (66.7)	2 (20.0)	1 (5.3)			

Grade I	29 (32.2)	6 (60.0)	10 (52.6)			

Grade II	1 (1.1)	2 (20.0)	8 (42.1)			

Charlson comorbidity index (%)						

0	47 (52.2)	5 (50.0)	9 (47.4)	0.727	0.923	0.761

1	25 (27.8)	2 (20.0)	6 (31.6)			

≥2	18 (20.0)	3 (30.0)	4 (21.1)			

Stair climbing				*0.004*	*<0.001*	0.417

4 or more	70 (77.8)	5 (50.0)	7 (36.8)			

Less than 4	20 (22.2)	5 (50.0)	12 (63.2)			

FEV1% (median, IQR)	85 (77-99)	95 (76-104)	71 (55-99)	0.493	0.173	0.133

Histology, n (%)				0.739	*0.003*	0.153

Adenocarcinoma	72 (80.0)	7 (70.9)	8 (42.1)			

Squamous cell cancer	18 (20.0)	3 (30.0)	11 (57.9)			

Tumor location				0.444	*0.026*	0.118

Upper or middle	6 (6.7)	-	4 (21.1)			

Distal or junction	84 (93.3)	10 (100)	15 (78.9)			

Sievert				0.471	0.295	0.072

I	39 (43.3)	3 (30.0)	10 (52.6)			

II	42 (46.7)	7 (70.0)	5 (26.3)			

III	3 (3.3)	-	-			

Oncological therapy				*<0.001*	*<0.001*	*0.015*

None	16 (17.8)	5 (50.0)	1 (5.3)			

Neoadjuvant chemotherapy	30 (33.3)	1 (10.0)	3 (15.8)			

Neoadjuvant chemoradiotherapy	44 (48.9)	1 (10.0)	4 (21.1)			

Other oncological treatments	-	3 (30.0)^1^	11 (57.9)^2^			

Surgical approach				0.885	-	-

McKeown	10 (11.1)	1 (10.0)				

Ivor Lewis	78 (86.7)	9 (90.0)				

Colon interposition	2 (2.2)	-				

Clinical Stage, n (%)				*<0.001*	0.867	*0.049*

I	17 (17.8)	2 (20.0)	3 (15.8)			

II	37 (41.1)	4 (40.0)	7 (36.8)			

III	36 (40.0)	2 (20.0)	9 (47.4)			

IV	-	2 (20.0)	-			

Gradus				0.273	*0.018*	0.483

1	11 (12.2)	3 (30.0)	7 (36.8)			

2	42 (46.7)	3 (30.0)	8 (42.1)			

3	16 (17.8)	3 (30.0)	4 (21.1)			

Pathological Stage, n (%)				0.777	-	-

0 (Complete response)	19 (21.1)	1 (10.0)				

I	22 (24.4)	3 (30.0)				

II	25 (27.8)	3 (30.0)				

III	24 (26.7)	3 (30.0)				

IV	-	-				

^1^Including two patients with prior oncological treatment due to synchronous liver metastases and one with persistent disease after definitive chemoradiotherapy.

^2^SeeTable 4. Including 7 patients with palliative radiotherapy and 4 patients with definitive chemoradiotherapy.

**Table 2:** Postoperative outcomes.

	Fit for any approach n=90	Fit for MIE only n=10	P value
Lymph nodes examined, median (IQR)	21 (17-28)	22 (9-28)	0.505

Pos. resection margins, n (%)	1 (1.1)	0	0.738

Complications, n (%)			

Any type	38 (42.2)	7 (70.0)	0.094

Minor (CDC Grades I-II)	32 (35.6)	2 (20.0)	0.325

Major (CDC Grades IIIa-V)	6 (6.7)	5 (50.0)	*<0.001*

Anastomotic leak	4 (10)	2 (20)	*0.049*

Pulmonary event	19 (21.1)	4 (40)	0.178

Cardiac event	12 (12)	1 (10)	0.766

Change in level of care, n (%)	0	3 (30.0)	*<0.001*

ICU stay, median (IQR)	1 (1-1)	1 (1-3)	0.196

Hospital stay, median (IQR)	9 (9-12)	10 (8-16)	0.542

Readmission rate within 30 days of discharge, n (%)	4 (4.4)	3 (30.0)	*0.003*

Comprehensive Complication Index, median (IQR)			

30-day	0 (0-20.9)	20.9 (0-92.2)	*0.032*

90-day	0 (0-20.9)	20.9 (0-92.7)	*0.038*

Mortality, n (%)			

30-day	0	1 (10.0)	*0.003*

90-day	0	2 (20.0)	*<0.001*

**Table 3:** Patients fit for MIE only.

Patient number	Age at surgery	Reason for inclusion as suitable for MIE only
Patient I	83	Clinical multilevel disease, age

Patient II	77	Obstructive pulmonary disease, synchronous lung cancer, pre-frailty

Patient III	59	Dilated cardiomyopathy, levosimendan treatment

Patient IV	68	Parkinson's disease, limited exercise capacity, BMI 38

Patient V	69	Synchronous liver metastases, remission with oncological treatment

Patient VI	84	Impaired pulmonary and renal function, age

Patient VII	59	Synchronous liver metastases, remission with oncological treatment

Patient VIII	83	Obstructive pulmonary disease, limited exercise capacity, age, pre-frailty

Patient IX	59	Persistent disease after definitive chemoradiotherapy, Child A liver cirrhosis, pre-frailty

Patient X	70	Child A liver cirrhosis, limited exercise capacity, BMI 36

**Table 4:** No surgery patients.

Reason for non-surgical therapy	Total of 19 patients
Physically unfit for surgery	7 patients

High-risk patient and unwilling for surgery	3 patients

High-risk patient with locally advanced disease considered conclusively unfit for surgery after neoadjuvant treatment	5 patients

High-risk patient with complete neoadjuvant response	2 patients

Child B liver cirrhosis	1 patient

Alcoholism	1 patient

## Data Availability

The data used to support the findings of this study are available from the corresponding author upon request.
